# The Efficacy and Safety of N-acetylcysteine and Taurine (Nefrosave®) in Chronic Kidney Disease: A Double-Blind, Multicenter, Placebo-Controlled Trial (DELAY-CKD)

**DOI:** 10.7759/cureus.105078

**Published:** 2026-03-11

**Authors:** AK Bhalla, Sampath Kumar, Himanshu Verma, Geeta Sheth, Bismay Kumar, Rajib Mondal, Gayatri V Jayaraman, Nagashankar N, Monjori Mitra

**Affiliations:** 1 Nephrology, Sir Ganga Ram Hospital, New Delhi, IND; 2 Nephrology, Meenakshi Mission Hospital, Madurai , IND; 3 Nephrology, VMMC & Safdarjung Hospital, New Delhi, IND; 4 Nephrology, JJ Hospital, Mumbai, IND; 5 Nephrology, Kumar Hypertension and Kidney Clinic, Kolkata, IND; 6 Nephrology, BMRC Hospital, Kolkata, IND; 7 Internal Medicine/Nephrology, Mass General Brigham Hospital System, Boston, USA; 8 Pharmacology, Fourrts India Laboratories, Chennai, IND; 9 Pediatrics, Institute of Child Health, Kolkata, IND

**Keywords:** chronic kidney disease, creatinine, cystatin c, estimated glomerular filtration rate, microalbuminuria, n-acetylcysteine, taurine, urinary albumin-creatinine ratio

## Abstract

Background and objective

Chronic kidney disease (CKD) is a progressive disorder with declining renal function and an elevated risk for end-stage renal disease (ESRD) and mortality. While oxidative stress is implicated in its pathogenesis, treatment using antioxidants remains uncertain. This study aimed to evaluate the effectiveness of the combination of N-acetylcysteine (NAC; 150 mg) and taurine (500 mg) in CKD.

Methods

A multicenter, double-blind, randomized, placebo-controlled trial was conducted across India between February 2021 and October 2023. Consenting adults aged 18-65 years with CKD stages 1-3 and serum creatinine <3.0 mg/dL were enrolled. Participants received NAC+taurine (Nefrosave^®^) or an identical placebo twice daily for 180 days, in addition to standard of care (SOC) and a low-protein diet. Primary endpoints included mean changes in urinary albumin-creatinine ratio (uACR), serum cystatin C, and estimated glomerular filtration rate (eGFR) from baseline to day 180. Secondary endpoints included time to event (hemodialysis, renal transplantation, treatment escalation). Safety was analyzed on the basis of adverse events (AEs).

Results

Efficacy analysis was conducted in 80 of the 100 randomized participants (NAC+taurine+SOC: 42; placebo+SOC: 38). Early-stage CKD (stages 1 and 2) was identified in 11 (26.19%) and 11 (28.95%) participants in the test and control arms, respectively. Microalbuminuria was observed in 10 (23.81%) participants in the NAC+taurine+SOC arm and 14 (36.84%) in the placebo+SOC arm. An overall 18.57% reduction in uACR at day 180 was observed with NAC+taurine supplementation compared with placebo, including a 34.09% reduction in microalbuminuria and a 19.34% reduction in macroalbuminuria; the between-group difference reached statistical significance for microalbuminuria (p=0.043). Overall, cystatin C decreased by 21.46% in the NAC+taurine+SOC arm, representing a 39.17% greater reduction than in the placebo+SOC arm (p=0.079). The increase in serum creatinine was lower in the NAC+taurine+SOC arm (0.03 mg/dL (1.78%)) compared with placebo+SOC (0.19 mg/dL (11.52%)), resulting in an overall relative reduction of 9.74% in the test arm. Comparable relative reductions were observed in CKD stages 1 and 2 (27.43%) and among participants with microalbuminuria (20.41%) in the NAC+taurine+SOC arm compared with placebo+SOC. Modest increases in mean eGFR were observed with NAC+taurine supplementation in CKD stage 3a, although overall changes were comparable to placebo+SOC. No clinically significant events were reported. Mild AEs were reported in five participants in the test arm.

Conclusions

Adjunctive NAC+taurine (Nefrosave^®^) therapy was well tolerated and was associated with favorable trends in albuminuria and other renal biomarkers as compared to SOC. The between-group difference in uACR reached statistical significance in participants with microalbuminuria and early CKD, supporting the need for further evaluation of this adjunctive strategy in larger trials.

## Introduction

Chronic kidney disease (CKD) is characterized by persistent structural or functional renal abnormalities lasting longer than three months that have adverse implications for an individual’s overall health [1]. CKD affects an estimated 850 million people worldwide [2] and is among the few categories of non-communicable diseases (NCDs) that continue to show rising age-standardized mortality rates [3]. The burden of CKD is disproportionately concentrated in low- and middle-income countries (LMICs) across Asia and Africa, where multiple disparities interact synergistically to drive disease prevalence and progression [4]. CKD is associated with reduced quality of life, increased economic burden, and an elevated risk of other NCDs, infectious diseases, mental health conditions, and neurocognitive impairment [5]. In the absence of timely detection and management, CKD may progress to end-stage renal disease (ESRD), at which point patients require lifelong dialysis or kidney transplantation, both of which remain costly and often inaccessible in developing countries [6]. Therefore, strategies aimed at addressing CKD in its earlier stages are crucial to slowing disease progression.

Several risk factors contribute to CKD pathogenesis through diverse mechanistic pathways, including oxidative stress-mediated glomerular, tubular, and endothelial injury [6-8]. This mechanism warrants particular attention because the kidney, as a highly metabolically active organ, is especially vulnerable to oxidative damage resulting from excess reactive oxygen species (ROS) generated during mitochondrial metabolic processes [7]. The imbalanced and self-perpetuating interplay between increased production of prooxidants and a diminished capacity of endogenous antioxidant defense systems is well recognized as a key driver of CKD progression [7,9,10]. Elevated levels of oxidative stress biomarkers are predictive of progression across successive CKD stages [11], further underscoring oxidative stress as a promising therapeutic target.

N-acetylcysteine (NAC), a precursor of L-cysteine and reduced glutathione, acts as a free radical scavenger. It has been reported to reduce serum creatinine levels [12] and slow the progression of CKD [13]. A systematic review demonstrated that treatment with NAC was associated with improvements in estimated glomerular filtration rate (eGFR) and serum creatinine levels compared with placebo [14]. Taurine, in contrast, is a β-amino acid with antioxidant properties that scavenges ROS and also functions as an intracellular osmolyte. It contributes to renal cellular homeostasis by regulating ion reabsorption and secretion, renal blood flow, osmoregulation, glomerular filtration, and urine composition, thereby exerting cytoprotective and renoprotective effects. Taurine supplementation has been shown to attenuate CKD progression and reduce hypertension and proteinuria [15,16].

The test combination of NAC (150 mg) and taurine (500 mg) has been reported to reduce urinary albumin-to-creatinine ratio (uACR) in microalbuminuric patients with type 2 diabetes [17,18], and has also been evaluated in nondiabetic individuals with early-stage CKD [19]. Although NAC and taurine are widely regarded as effective nutraceuticals, their therapeutic role in CKD has not been comprehensively characterized. The DELAY-CKD trial assessed the efficacy of antioxidant therapy with NAC+taurine (Nefrosave®) administered in conjunction with standard of care (SOC) and a low-protein diet in patients with CKD stages 1 to 3.

## Materials and methods

Study setting

The DELAY-CKD trial was a multicenter, double-blind, randomized, placebo-controlled study conducted between February 2021 and October 2023 at the following six sites in India: Sir Ganga Ram Hospital (New Delhi), Meenakshi Mission Hospital and Research Centre (Madurai, Tamil Nadu), Grant Government Medical College and JJ Group of Hospitals (Mumbai), Kumar Hypertension & Kidney Clinic (Howrah, West Bengal), BMRC Hospitals (Barrackpore, West Bengal), and VMMC and Safdarjung Hospital (New Delhi). The trial was prospectively registered with the Clinical Trials Registry-India (CTRI) on April 3, 2020 (Reference number: CTRI/2020/04/024476).

Study objectives and endpoints

The primary objective of this study was to evaluate the comparative effectiveness of NAC+taurine+SOC versus placebo+SOC in delaying CKD progression. To address this, the primary endpoints were changes from baseline to day 180 in uACR, serum cystatin C, and eGFR (assessed by the CKD Epidemiology Collaboration or CKD-EPI creatinine equation). The secondary objective was to evaluate the time to clinically relevant events, comprising initiation of hemodialysis, renal transplantation, or significant modification of renal management. Safety was assessed based on the incidence of adverse events (AEs). Serum creatinine levels were measured as an additional indicator of renal function. All laboratory analyses were conducted at a central laboratory to ensure consistency across the study. Vital signs (blood pressure, temperature, pulse rate, and respiratory rate) were measured for all participants.

In addition to planned endpoints, analysis was performed in subgroups of participants stratified by CKD stage (stage 1 and 2: eGFR ≥60 mL/min/1.73 m²; stage 3a: eGFR 45-59 mL/min/1.73 m²; stage 3b: eGFR 30-44 mL/min/1.73 m²) or baseline uACR (A1: <30 µg/mg; A2: 30-300 µg/mg (microalbuminuria); A3: >300 µg/mg (macroalbuminuria)) [20].

Eligibility criteria and intervention allocation

Adults aged 18-65 years with CKD stages 1-3, defined according to National Kidney Foundation-Kidney Disease Outcomes Quality Initiative (NKF-KDOQI) criteria, were included in the trial, provided their serum creatinine was <3.0 mg/dL. Key exclusion criteria included type 1 diabetes mellitus, pregnancy or lactation, advanced CKD (stage 4 or above), renal allograft, recent myocardial infarction or coronary procedure, New York Heart Association (NYHA) class III/IV heart failure, known intolerance to NAC or taurine, untreated urinary tract infection, or comorbidities potentially confounding proteinuria assessment. Individuals currently undergoing dialysis and those with BMI <18 or >30 kg/m², significant systemic illness, and abnormal laboratory parameters that could impede study participation were also excluded from the study. Concurrent enrollment in a different trial was prohibited.

Eligible participants were randomized in a 1:1 ratio using block randomization with a computer-generated sequence to receive 150 mg NAC + 500 mg taurine (Nefrosave®, Fourrts India Laboratories) or an identical placebo twice daily for 180 days, in addition to SOC. All participants were advised to adhere to a low-protein diet. Study drugs and placebo were identical in appearance and administered in a double-blind manner, whereby participants, investigators, and study staff remained blinded to allocation. Study visits were scheduled at baseline and at days 30, 90, and 180 (±7 days). Compliance with the study medication regimen was tracked using medication accountability logs and capsule counts at each visit.

Sample size calculation and statistical analysis

Sample size was determined based on an expected effect size of 10% in uACR between the control and intervention groups. To detect the pre-specified difference with an α of 0.05 (Z_α_ = 1.96) and power (1-β) of 80%, 41 participants per arm were calculated based on the formula n > 2(Z_α_ + Z_1-β_)² × SD²/d². Assuming a 20% attrition rate during follow-up, 50 participants were enrolled in each arm. To better evaluate efficacy under optimal adherence to the randomized group and completion of long-term follow-up across all study visits, per protocol (PP) analysis was implemented.

Statistical analyses were conducted using IBM SPSS Statistics 29.0.2.0 (IBM Corp., Armonk, NY) in accordance with ICH E9 statistical principles for clinical trials. Repeated measures ANOVA with post-hoc (Bonferroni) was used to compare the two groups across the visits, and the Welch t-test was used to compare the mean change between the two groups. The proportion of participants was compared using the chi-square test. Median imputation was done for missing values. A two-sided p-value <0.05 was considered statistically significant, unless otherwise specified.

To obtain robust estimates of uncertainty in the observed treatment effect (difference in mean change of cystatin C), a nonparametric bootstrap procedure was performed. Specifically, we generated 1,000 bootstrap replications by sampling with replacement for the overall patients and at each stage. In each bootstrap sample, we recalculated the difference in mean change, creating an empirical distribution of the differences. The 95% confidence interval (CI) was derived using the percentile method. The empirical two-sided p-value was calculated as:

$2 \times \min\bigl(P(\Delta \le 0),\, P(\Delta \ge 0)\bigr)$

where ∆=mean(nefrosave)-mean(placebo).

Ethical considerations

This study was conducted in accordance with the principles of Declaration of Helsinki, Ethical Guidelines for Biomedical Research on Human Subjects issued by the Indian Council of Medical Research (ICMR), as well as in compliance with the requirements of the clinical study protocol approved by the Ethics Committees of study sites (Ethics Committee Sir Ganga Ram Hospital (ECR/20/Inst/DL/2013/RR-19; New Delhi), Institutional Ethics Committee for Meenakshi Mission Hospital and Research Centre (ECR/398/Inst/TN/2013/RR-19; Tamil Nadu), Institutional Ethics Committee for Grant Government Medical College and JJ Group of Hospitals (ECR/382/Inst/MH/2013/RR-19; Mumbai), OrciVita Independent Ethics Committee and Hurip Independent Bioethics Committee (ECR/335/Indt/WB/2020 and ECR/103/Indt/WB/2013/RR-19, respectively; West Bengal), and Institutional Ethics Committee for VMMC and Safdarjung Hospital (ECR/593/Inst/DL/2014/RR-20; New Delhi). Written informed consent was obtained from all participants before study enrollment.

## Results

Participant disposition

**Figure 1:**
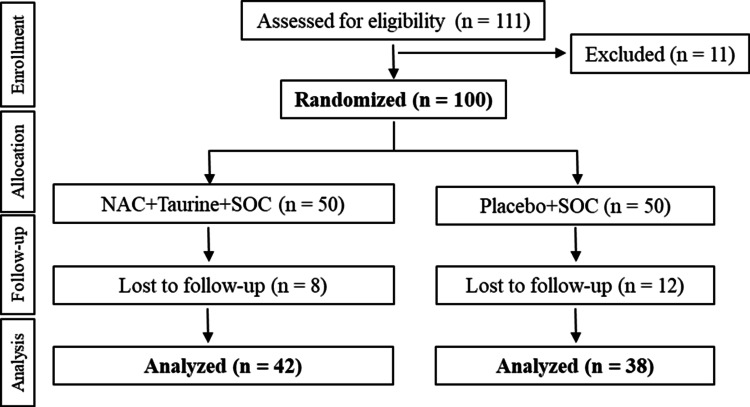
Participant disposition NAC: N-acetylcysteine; SOC: standard of care

Of the 111 patients screened, 100 were eligible and randomly assigned in a 1:1 ratio to receive NAC+taurine+SOC (test; n=50) or placebo+SOC (control; n=50). Twenty participants (NAC+taurine+SOC: 8; placebo+SOC: 12) withdrew before day 180 follow-up visit due to withdrawal of consent or loss to follow-up. The remaining 80 participants (NAC+taurine+SOC: 42; placebo+SOC: 38) comprised the PP efficacy population (Figure 1).

Baseline characteristics of participants

The mean age of the PP participants was 52.88 years in the test and 53.95 years in the control. Most PP participants were diagnosed with stage 3 CKD (NAC+taurine+SOC: 31 (73.81%); placebo+SOC: 27 (71.05%)). CKD stage 1 and 2 were diagnosed in 11 (26.19%) and 11 (28.95%) participants in the test and control arm, respectively. Classified by uACR levels of the PP participants, 10 (23.81%) in the test arm and 14 (36.84%) in the control arm had microalbuminuria. Macroalbuminuria was detected in 19 (45.24%) participants in the test and 13 (34.21%) in the control arm. Type 2 diabetes mellitus and hypertension were the predominant comorbidities. Baseline characteristics were comparable between the study arms (Table 1).

**Table 1:** Baseline characteristics of participants NAC: N-acetylcysteine; SOC: standard of care; BMI: body mass index; SD: standard deviation; CKD: chronic kidney disease; uACR: urinary albumin-creatinine ratio

	NAC+taurine+SOC (n = 42)	Placebo+SOC (n = 38)	P-value
Age, years, mean ± SD	52.88 ± 10.72	53.95 ± 10.74	0.658
BMI kg/m^2^, mean ± SD	24.62 ± 3.52	24.64 ± 2.58	0.975
Sex, n (%)			
Male	26 (61.90%)	27 (71.05%)	0.388
Female	16 (38.10%)	11 (28.95%)	
Stage of CKD, n (%)			
Stage 1 and 2	11 (26.19%)	11 (28.95%)	0.783
Stage 3a	11 (26.19%)	11 (28.95%)	0.783
Stage 3b	20 (47.62%)	16 (42.11%)	0.621
uACR level, n (%)			
<30 µg/mg	13 (30.95%)	11 (28.95%)	0.845
30-300 µg/mg	10 (23.81%)	14 (36.84%)	0.204
>300 µg/mg	19 (45.24%)	13 (34.21%)	0.315
Comorbid conditions, n (%)			
Type 2 diabetes mellitus	14 (33.33%)	13 (34.21%)	0.934
Hypertension	18 (42.86%)	21 (55.26%)	0.512

Mean change in uACR from baseline to day 180

**Figure 2:**
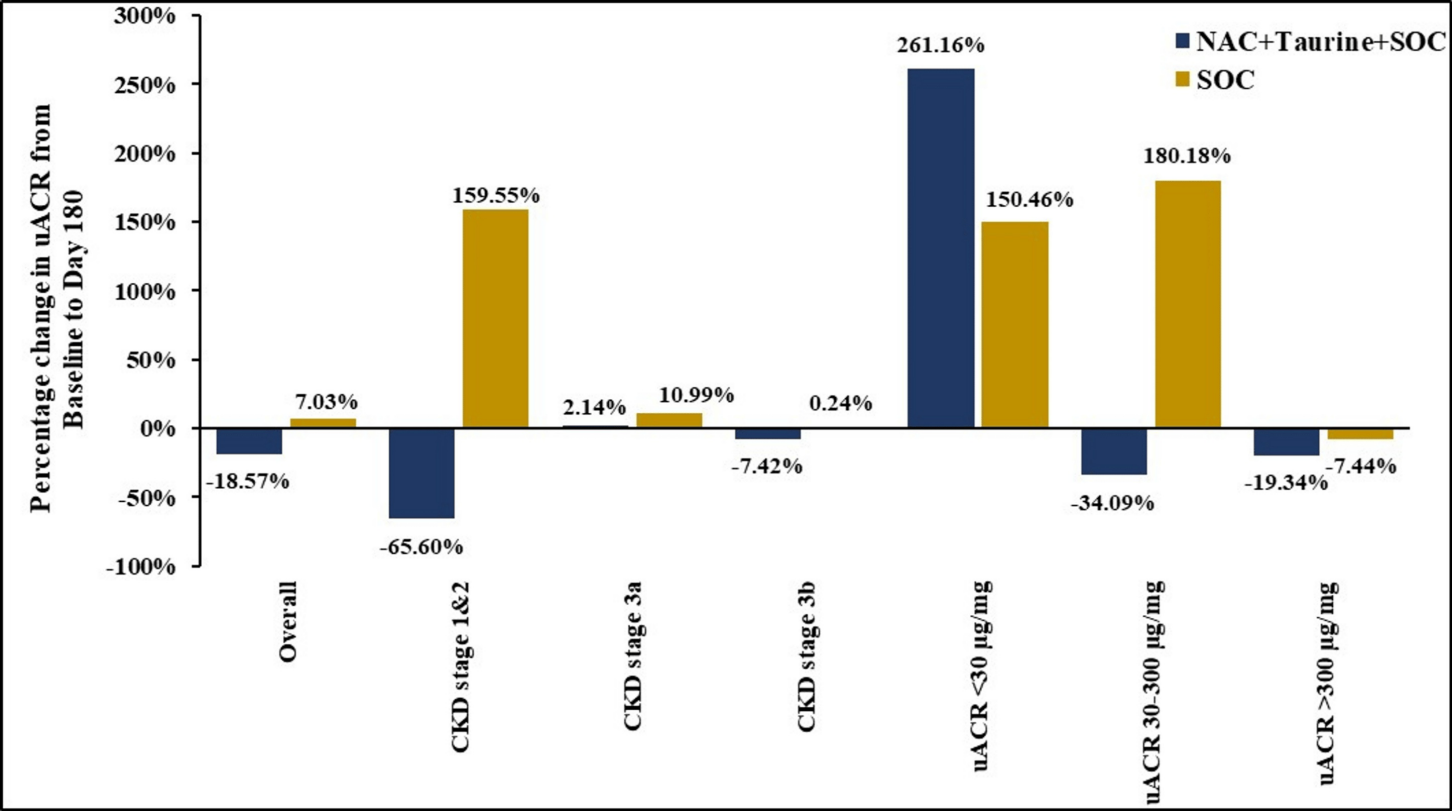
Percentage change in uACR from baseline to day 180 uACR: urinary albumin-creatinine ratio; NAC: N-acetylcysteine; SOC: standard of care; CKD: chronic kidney disease

Baseline uACR was higher in the test arm (858.95 μg/mg) compared to the control (608.91 μg/mg). At day 180, a notable divergent trend emerged, wherein the test arm demonstrated a reduction in mean uACR by 18.57% (159.50 µg/mg), contrasting with a corresponding increase in the control arm by 7.03% (42.79 µg/mg). Although the between-group difference did not attain statistical significance (p=0.321), the opposing directional trend favored the test arm with an expected decrease observed in uACR (Figure 2, Table 2).

**Table 2:** Mean change in uACR (µg/mg) from baseline to day 180 ^*^Statistically significant uACR: urinary albumin-creatinine ratio; NAC: N-acetylcysteine; SOC: standard of care; SD: standard deviation; PP: per protocol; CKD: chronic kidney disease

	Baseline	Day 180	Baseline	Day 180	Mean difference from baseline to day 180, Δ (Δ%)		P-value
	NAC+taurine+SOC, mean ± SD		Placebo+SOC, mean ± SD		NAC+taurine	Placebo+SOC	
Overall (PP)	858.95 ± 1478.94	699.45 ± 1243.31	608.91 ± 1146.24	651.70 ± 1405.98	-159.50 (-18.57%)	42.79 (7.03%)	0.321
Stratified by CKD stage							
CKD stage 1 and 2	791.19 ± 1536.25	272.16 ± 586.95	62.60 ± 66.15	162.48 ± 409.62	-519.03 (-65.60%)	99.88 (159.55%)	0.079
CKD stage 3a	991.03 ± 1920.93	1012.20 ± 1652.37	400.67 ± 407.35	444.70 ± 479.83	21.17 (2.14%)	44.03 (10.99%)	0.914
CKD stage 3b	823.58 ± 1233.53	762.46 ± 1245.35	1127.66 ± 1609.23	1130.32 ± 2039.48	-61.12 (-7.42%)	2.66 (0.24%)	0.869
Stratified by baseline uACR							
<30 µg/mg (A_1_)	12.77 ± 6.31	46.12 ± 79.32	8.62 ± 5.09	21.59 ± 34.16	33.35 (261.16%)	12.97 (150.46%)	0.446
30-300 µg/mg (A_2_)	126.97 ± 71.72	83.69 ± 89.28	121.71 ± 71.49	341.01 ± 407.05	-43.28 (-34.09%)	219.30 (180.18%)	0.043^*^
>300µg/mg (A_3_)	1823.18 ± 1784.19	1470.55 ± 1538.28	1641.52 ± 1511.01	1519.45 ± 2149.96	-352.63 (-19.34%)	-122.07 (-7.44%)	0.653

The opposing trend in uACR trajectories between the study arms in overall analysis prompted stratification and indicated the need to delineate potential CKD stage- or albuminuria-specific responses within subgroups of participants with these specificities (Table 1). A decrease in uACR by 65.60% (519.03 µg/mg) was observed among participants with CKD stage 1 and 2 upon treatment with NAC+taurine+SOC. In contrast, participants treated with placebo+SOC showed an increase in uACR by 159.55% (99.88 µg/mg) with p-value approaching significance (p=0.079), again emphasizing the beneficial outcome when NAC+taurine was supplemented to SOC (Table 2, Figure 2).

NAC+taurine supplementation was associated with a decrease in uACR by 34.09% (43.28 µg/mg) among participants with microalbuminuria and by 19.34% (352.63 µg/mg) among participants with macroalbuminuria, concomitant to a corresponding increase or lower decrease, respectively, in the control arm. The between-group difference was statistically significant for microalbuminuric participants (p=0.043), reiterating the advantage of NAC+Taurine supplementation (Table 2, Figure 2).

Mean change in serum cystatin C from baseline to day 180

**Figure 3:**
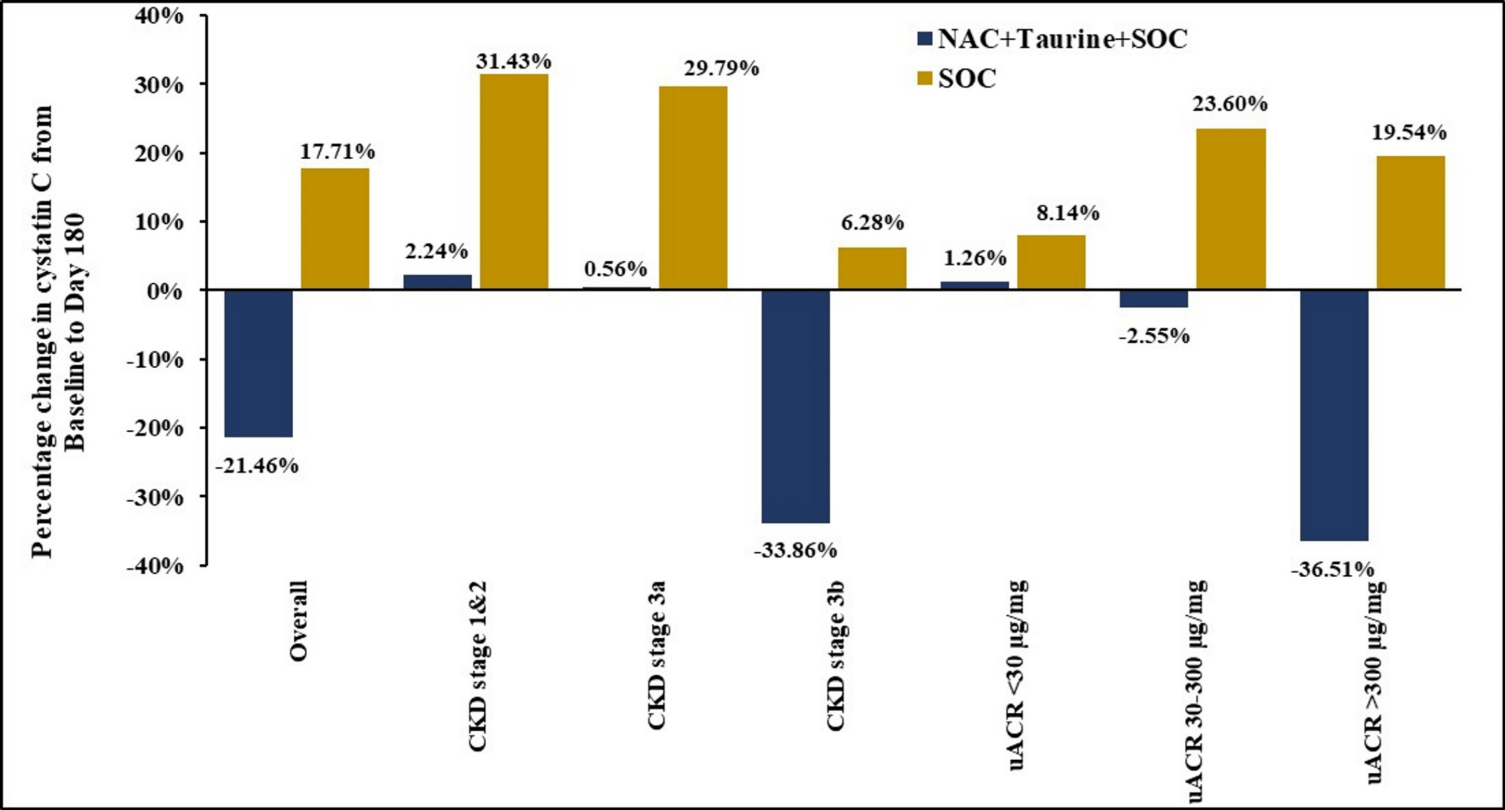
Percentage change in serum cystatin C from baseline to day 180 NAC: N-acetylcysteine; SOC: standard of care; CKD: chronic kidney disease; uACR: urinary albumin-creatinine ratio

The mean serum cystatin C levels in the test and control arms in the overall and stratified cohorts are outlined in Figure 3 and Table 3. In the PP population, baseline mean serum cystatin C was comparatively higher in the test arm (2.33 mg/dL) than that in the control (1.75 mg/dL). By day 180, however, the trend reversed with cystatin C levels attaining 1.83 mg/dL and 2.06 mg/dL in the test and control arm, respectively. Overall, the test arm exhibited a decrease in mean serum cystatin C levels by 21.46% (0.50 mg/dL) while the control arm showed an increase by 17.71% (0.31 mg/dL), with a between-group difference of 39.17% approaching significance (p=0.079). Bootstrapping demonstrated a statistically significant between-group difference (p=0.004) in the overall analysis as well as in participants with CKD stage 3a (p=0.038) or 3b (p=0.044). The attenuation of cystatin C levels hints at the potential of NAC+taurine supplementation to stabilize glomerular filtration dynamics, perhaps in advanced disease where baseline impairment is most pronounced. This was also corroborated by the finding that the between-group differences were statistically significant upon bootstrapping in participants with microalbuminuria (p=0.044) or macroalbuminuria (p=0.012).

**Table 3:** Mean change in serum cystatin C (mg/dL) from baseline to day 180 ^*^Statistically significant NAC: N-acetylcysteine; SOC: standard of care; SD: standard deviation; PP: per protocol; CKD: chronic kidney disease; uACR: urinary albumin-creatinine ratio

	Baseline	Day 180	Baseline	Day 180	Mean difference from baseline to day 180, Δ (Δ%)		P-value	Bootstrapping resampling size=1000	
	NAC+taurine+SOC, mean ± SD		Placebo+SOC, mean ± SD		NAC+taurine+SOC	Placebo+SOC		Mean difference (between-group)	P-value
Overall (PP)	2.33 ± 2.80	1.83 ± 0.60	1.75 ± 0.63	2.06 ± 0.89	-0.50 (-21.46%)	0.31 (17.71%)	0.079	-0.80	0.004^*^
Stratified by CKD stage									
CKD stage 1 and 2	1.34 ± 0.53	1.37 ± 0.32	1.40 ± 0.22	1.84 ± 1.02	0.03 (2.24%)	0.44 (31.43%)	0.241	-0.42	0.182
CKD stage 3a	1.77 ± 0.52	1.78 ± 0.52	1.41 ± 0.62	1.83 ± 0.78	0.01 (0.56%)	0.42 (29.79%)	0.069	-0.43	0.038^*^
CKD stage 3b	3.19 ± 3.88	2.11 ± 0.62	2.23 ± 0.52	2.37 ± 0.73	-1.08 (-33.86%)	0.14 (6.28%)	0.225	-1.16	0.044^*^
Stratified by baseline uACR									
<30 µg/mg (A_1_)	1.59 ± 0.54	1.61 ± 0.53	1.72 ± 0.59	1.86 ± 0.63	0.02 (1.26%)	0.14 (8.14%)	0.651	-0.11	0.640
30-300 µg/mg (A_2_)	1.96 ± 0.68	1.91 ± 0.67	1.78 ± 0.56	2.20 ± 1.03	-0.05 (-2.55%)	0.42 (23.60%)	0.159	-0.47	0.044^*^
>300µg/mg (A_3_)	3.04 ± 4.05	1.93 ± 0.61	1.74 ± 0.78	2.08 ± 0.85	-1.11 (-36.51%)	0.34 (19.54%)	0.207	-1.46	0.012^*^

Mean change in serum creatinine from baseline to day 180

**Figure 4:**
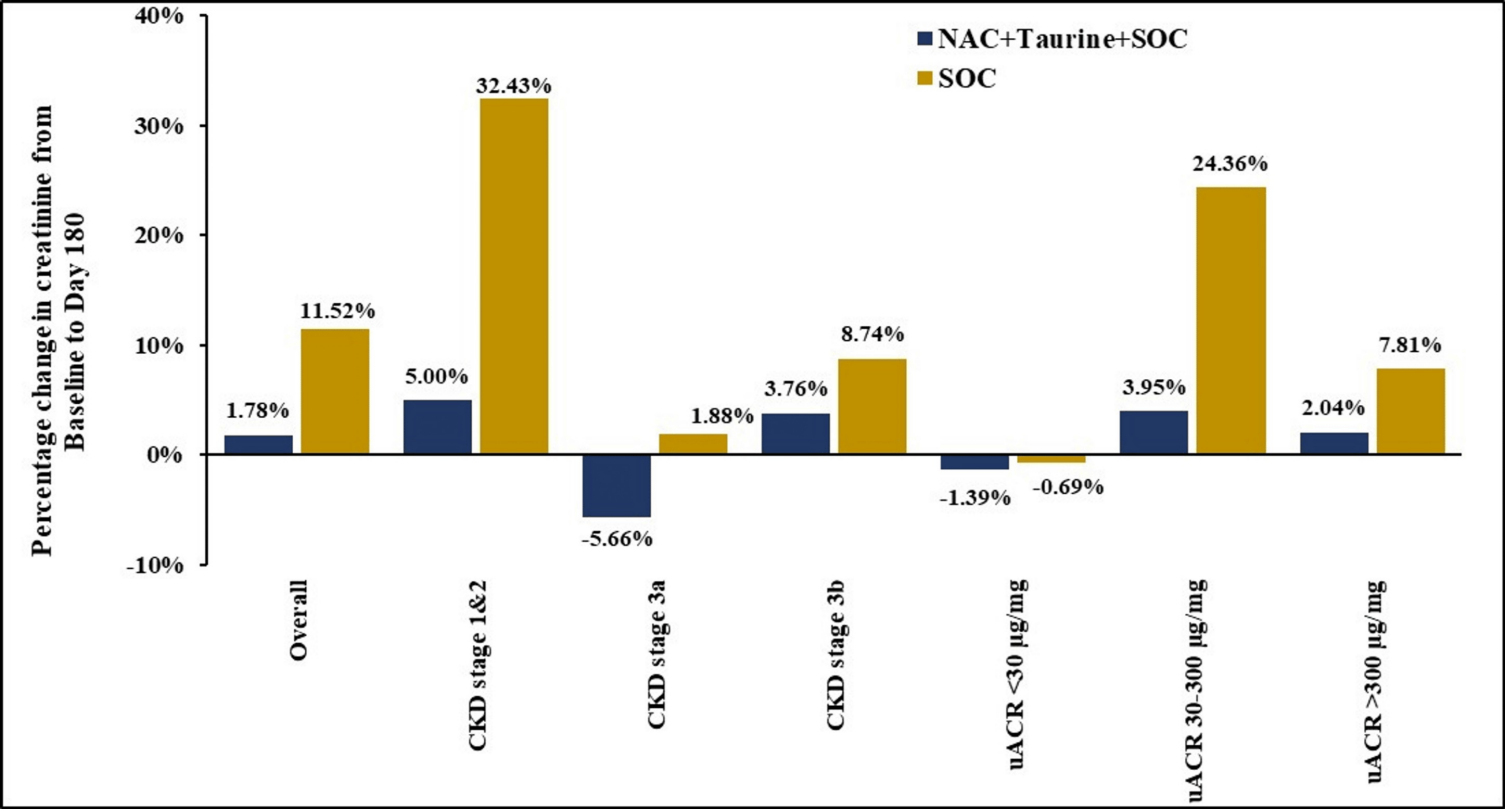
Percentage change in serum creatinine from baseline to day 180 NAC: N-acetylcysteine; SOC: standard of care; CKD: chronic kidney disease; uACR: urinary albumin-creatinine ratio

Longitudinal assessment revealed an overall increase in mean serum creatinine levels in the PP population of both arms, as evidenced in Figure 4 and Table 4. The magnitude of increase, however, was considerably smaller in the test arm (0.03 mg/dL (1.78%) compared to the control (0.19 mg/dL (11.52%), despite both groups sharing similar creatinine values at baseline (test: 1.69 mg/dL; control: 1.65 mg/dL). Although between-group differences were not statistically significant, relative reductions of 9.74% in the overall analysis, 27.43% in participants with CKD stages 1 and 2, and 20.41% in participants with microalbuminuria were observed in the test arm compared with the control arm. In participants with CKD stage 3a, mean serum creatinine levels were found to decrease by 0.09 mg/dL (5.66%) in the test arm, corresponding to an increase by 0.03 mg/dL (1.88%) in the control arm. Together, these findings suggest that the addition of NAC+taurine to SOC might be a useful adjunctive therapy for CKD.

**Table 4:** Mean change in serum creatinine (mg/dL) from baseline to day 180 NAC: N-acetylcysteine; SOC: standard of care; SD: standard deviation; PP: per protocol; CKD: chronic kidney disease; uACR: urinary albumin-creatinine ratio

	Baseline	Day 180	Baseline	Day 180	Mean difference from baseline to day 180, Δ (Δ%)		P-value
	NAC+taurine+SOC, mean ± SD		Placebo+SOC, mean ± SD		NAC+taurine+SOC	Placebo+SOC	
Overall (PP)	1.69 ± 0.63	1.72 ± 0.67	1.65 ± 0.54	1.84 ± 0.88	0.03 (1.78%)	0.19 (11.52%)	0.229
Stratified by CKD stage							
CKD stage 1 and 2	1.00 ± 0.25	1.05 ± 0.19	1.11 ± 0.27	1.47 ± 0.98	0.05 (5.00%)	0.36 (32.43%)	0.267
CKD stage 3a	1.59 ± 0.53	1.50 ± 0.42	1.60 ± 0.38	1.63 ± 0.52	-0.09 (-5.66%)	0.03 (1.88%)	0.456
CKD stage 3b	2.13 ± 0.43	2.21 ± 0.55	2.06 ± 0.41	2.24 ± 0.88	0.08 (3.76%)	0.18 (8.74%)	0.677
Stratified by baseline uACR							
<30 µg/mg (A_1_)	1.44 ± 0.43	1.42 ± 0.44	1.45 ± 0.44	1.44 ± 0.48	-0.02 (-1.39%)	-0.01 (-0.69%)	0.986
30-300 µg/mg (A_2_)	1.52 ± 0.69	1.58 ± 0.71	1.56 ± 0.52	1.94 ± 0.91	0.06 (3.95%)	0.38 (24.36%)	0.256
>300µg/mg (A_3_)	1.96 ± 0.63	2.00 ± 0.69	1.92 ± 0.56	2.07 ± 1.03	0.04 (2.04%)	0.15 (7.81%)	0.640

Mean change in eGFR from baseline to day 180

**Figure 5:**
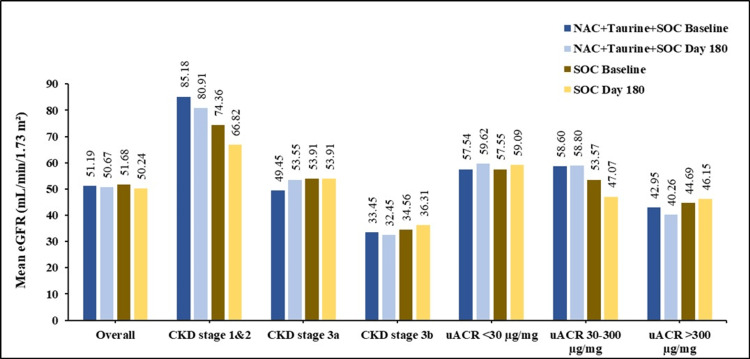
Mean eGFR at baseline and day 180 eGFR: estimated glomerular filtration rate; NAC: N-acetylcysteine; SOC: standard of care; CKD: chronic kidney disease; uACR: urinary albumin-creatinine ratio

eGFR values across treatment arms are summarized in Figure 5 and Table 5. In the overall cohort, mean eGFR remained almost unchanged between baseline and day 180 in both the test and the control arms. The marginal decline observed in the test arm (-0.52 mL/min/1.73 m²) was numerically smaller than that in the control arm (-1.44 mL/min/1.73 m²). An important observation was that the mean eGFR in CKD stage 3a increased from 49.45 to 53.55 mL/min/1.73 m² in the test arm, while it remained unchanged in the control arm (p=0.510 for between-group difference). Also, the baseline mean eGFR in microalbuminuric participants was maintained at day 180 in the test arm (58.60 vs. 58.80 mL/min/1.73 m²), corresponding to a decrease in the control arm (53.57 vs. 47.07 mL/min/1.73 m²; p=0.266 for between-group difference).

**Table 5:** Mean change in eGFR from baseline to day 180 eGFR: estimated glomerular filtration rate; NAC: N-acetylcysteine; SOC: standard of care; SD: standard deviation; PP: per protocol; CKD: chronic kidney disease; uACR: urinary albumin-creatinine ratio

	Baseline	Day 180	Baseline	Day 180	Mean difference from baseline to day 180, Δ (Δ%)		P-value
	NAC+taurine+SOC, mean ± SD		Placebo+SOC, mean ± SD		NAC+taurine+SOC	Placebo+SOC	
Overall (PP)	51.19 ± 25.15	50.67 ± 25.12	51.68 ± 20.38	50.24 ± 23.69	-0.52 (-1.02%)	-1.44 (-2.79%)	0.785
Stratified by CKD stage							
CKD stage 1 and 2	85.18 ± 19.84	80.91 ± 16.23	74.36 ± 15.12	66.82 ± 27.21	-4.27 (-5.01%)	-7.54 (-10.14%)	0.665
CKD stage 3a	49.45 ± 15.24	53.55 ± 22.89	53.91 ± 14.31	53.91 ± 14.49	4.10 (8.29%)	0.00 (0.00%)	0.510
CKD stage 3b	33.45 ± 5.77	32.45 ± 7.96	34.56 ± 6.49	36.31 ± 18.23	-1.00 (-2.99%)	1.75 (5.06%)	0.558
Stratified by baseline uACR							
<30 µg/mg (A_1_)	57.54 ± 19.34	59.62 ± 23.04	57.55 ± 16.93	59.09 ± 18.59	2.08 (3.61%)	1.54 (2.68%)	0.929
30-300 µg/mg (A_2_)	58.60 ± 32.39	58.80 ± 34.29	53.57 ± 24.44	47.07 ± 28.02	0.20 (0.34%)	-6.50 (-12.13%)	0.266
>300 µg/mg (A_3_)	42.95 ± 23.13	40.26 ± 16.93	44.69 ± 17.50	46.15 ± 22.14	-2.69 (-6.26%)	1.46 (3.27%)	0.485

Time-to-event and adverse events

During the study, no participant in either arm experienced any clinical event necessitating hemodialysis, renal transplantation, or escalation of their existing treatment regimen. This absence of progression-related endpoints was consistent in both arms and suggests relative clinical stability. AEs were reported in a total of six out of 100 enrolled participants (6.00%). Of these, five events occurred in the test arm, with all affected individuals recovering fully without sequelae. These AEs comprised mild symptoms, including vomiting, abdominal pain, common cold, sore throat, weakness, vertigo, fever, and swelling and tingling. Of these, only swelling and tingling were adjudicated as likely or probably related to the study medication, while the rest were deemed unrelated. No AE was reported in the control arm. Collectively, these findings highlight the favorable safety and tolerability profile of NAC+taurine supplementation, with no serious or persistent drug-related complications identified over the course of the study.

## Discussion

Slowing progression to ESRD remains a primary objective of CKD management, often requiring a combination of therapeutic strategies. Clinical guidelines recommend initiating renin-angiotensin system inhibitors (RASi) (angiotensin-converting enzyme inhibitors (ACEi), or angiotensin II receptor blockers (ARB)) in individuals with CKD and severely increased albuminuria [1]. Adjunctive antioxidant supplementation may offer further benefit. Although there is a strong biological rationale linking oxidative stress to CKD progression and extra-renal complications, clinical data supporting the effectiveness of antioxidant therapy have yielded inconsistent results. In an updated systematic review and meta-analysis of 95 studies involving more than 10,000 participants, investigators found that antioxidant supplementation was associated with a slower decline in eGFR and stabilization of creatinine levels, but showed no significant impact on uACR or the progression of albuminuria. While the review concluded that antioxidant supplementation may reduce cardiovascular events and slow kidney disease progression [21], the presence of multiple conflicting findings underscores the complexity of antioxidant intervention and provides the much-needed context for the present study.

Results from the DELAY-CKD trial show that adjunctive NAC+taurine therapy not only provides expected overall outcomes but also elicits albuminuria- and stage-dependent nephroprotective effects. Analysis of these subgroups is a salient feature of the study. The preponderance of participants with CKD stage 3 in the current cohort was perhaps due to the relatively late diagnosis of the disease in routine clinical practice. Overall analysis revealed statistically comparable inter-arm differences for overall changes in uACR, serum creatinine, and eGFR from baseline to day 180. Nevertheless, the observed decline in eGFR and rise in serum creatinine were numerically smaller upon NAC+taurine supplementation compared to SOC, leading to beneficial relative reductions over SOC alone.

More importantly, there was an 18.57% overall decrease in uACR upon supplementation with NAC+taurine, while placebo+SOC resulted in an overall increase in uACR. Notably, there was a 65.60% and 34.09% decrease in uACR, respectively, among stages 1 and 2 and microalbuminuric participants receiving NAC+taurine. Corresponding participants treated with placebo+SOC displayed very high increases in uACR of the magnitude 159.55% and 180.18%, respectively. Thus, SOC alone may not be sufficient for the management of microalbuminuria. These differential responses suggest that NAC+taurine may be effective in CKD, particularly in early stages of albuminuric CKD, potentially offering a window of therapeutic opportunity before the development of more advanced nephropathy.

Indeed, this hypothesis was supported by findings in patients with macroalbuminuria, in whom uACR levels declined by 19.34%, whereas a greater 34.09% reduction was observed in those with microalbuminuria, reinforcing the notion that NAC+taurine therapy may confer more modest effects on uACR in advanced versus early-stage CKD. Notably, mean eGFR demonstrated a slight increase in patients with CKD stage 3a receiving NAC+taurine, while it remained unchanged in the placebo+SOC group. Furthermore, Sengupta et al. reported significant improvements in eGFR among individuals with CKD stage 3a or more advanced disease following NAC+taurine therapy [19]. These findings suggest that the incremental benefit of NAC+taurine over standard of care may be more evident in later stages of CKD, although this needs further validation.

The observations with the relatively unexplored renal marker, serum cystatin C, unveil a different perspective. Overall, mean cystatin C levels rose by 17.71% upon placebo+SOC treatment. In contrast, NAC+taurine supplementation led to a decline by 21.46%, revealing a significant between-group difference upon bootstrapping. Similar significance was obtained in subgroups of participants with CKD stage 3, microalbuminuria, and macroalbuminuria, although an appreciable decrease above 30% was evident only upon supplementation in stage 3b or macroalbuminuria. Taken together, the current results support a crucial role for NAC+taurine supplementation in managing uACR during early stages and perhaps cystatin C in advanced stages of the disease. However, a systematic review by Huang et al. (2021) reported no change in cystatin C after NAC administration [12], highlighting the need for more evidence in this regard.

Our results align with a mixed body of evidence on the efficacy of antioxidant therapy in CKD. Individual trials on NAC supplementation have often found little to no benefit on renal biomarkers, while pooled data suggest positive trends. On the other hand, the nephroprotective effects of taurine have been extensively documented in experimental models, but evidence supporting its role in CKD management in humans remains lacking. In a randomized trial of non‐diabetic CKD patients, high-dose oral NAC did not affect proteinuria or tubular injury markers [22], while pooled data from 15 randomized controlled trials found that NAC can slightly improve serum creatinine and eGFR compared to placebo [10]. Several other clinical studies showed that short-term oral NAC administration in stage 3 CKD patients did not yield significant improvements in renal function or proteinuria levels, regardless of diabetic status [23].

However, the significant benefits in microalbuminuric patients observed in our trial are echoed by previous studies. Among patients with type 2 diabetes and microalbuminuria, NAC+taurine supplementation was associated with a reduction in the levels of transforming growth factor-beta (TGF-b) and uACR [13,14]. Collectively, these reinforce that the benefits of antioxidant therapy in CKD remain uncertain and likely dependent upon patient selection, disease stage, metabolic milieu, dosing regimens, and adjunctive therapies. The modest but consistent trends favoring NAC+taurine, especially in early albuminuric CKD in our study, align with this nuanced landscape. These highlight the need for future investigations to optimize therapeutic strategies tailored to CKD phenotypes and biochemical profiles, while incorporating sufficient treatment duration and robust endpoints to capture meaningful clinical outcomes. This is of importance, given that clinical trials of antioxidants other than NAC and taurine also struggled to show clear benefits among diverse CKD patient populations [20,24-30].

The uniform trends of improvement in albuminuria, lesser increase in creatinine levels, and lesser decline in glomerular filtration in the overall analysis point to nuanced nephroprotective effects of dual antioxidant supplementation. Non-significant findings, despite consistent directional trends in our study, might also be explained by patient heterogeneity, which may have diluted treatment effects that might be more pronounced in specific subgroups. In fact, subgroup analyses based on baseline albuminuria levels and disease stage helped us deduce important conclusions from the current dataset. The relatively small sample size, however, limited our ability to detect modest but clinically meaningful effects. The drop in sample size, especially due to patients lost to follow-up, was partly attributed to the then ongoing COVID-19 pandemic. Another limitation of the study is the lack of control over the low-protein diet that participants were advised to follow, since this was not formally verified. Future studies should preferably be conducted in larger cohorts followed over a longer duration to better conclude on disease progression.

## Conclusions

Adjunctive NAC+taurine (Nefrosave®) therapy was associated with favorable trends in albuminuria and other renal biomarkers as compared to SOC alone. The statistically significant between-group difference in uACR, coupled with an excellent safety profile, suggests that NAC+taurine supplementation may have clinical utility, particularly in patients with microalbuminuria and early-stage CKD. Further evaluation of this adjunctive strategy in larger trials with longer follow-up is warranted. Overall, it may be advantageous for nephrologists to prioritize early intervention, and integrating NAC plus taurine with standard-of-care treatment and a low-protein diet could help optimize renal outcomes and overall patient management.
